# Enhancement of Canonical Wnt/β-Catenin Signaling Activity by HCV Core Protein Promotes Cell Growth of Hepatocellular Carcinoma Cells

**DOI:** 10.1371/journal.pone.0027496

**Published:** 2011-11-15

**Authors:** Jiao Liu, Xiong Ding, Jia Tang, Youde Cao, Peng Hu, Fan Zhou, Xiaoliang Shan, Xuefei Cai, Qingmei Chen, Ning Ling, Bingqiang Zhang, Yang Bi, Ke Chen, Hong Ren, Ailong Huang, Tong-Chuan He, Ni Tang

**Affiliations:** 1 The Second Affiliated Hospital and the Key Laboratory of Molecular Biology of Infectious Diseases designated by the Chinese Ministry of Education, Chongqing Medical University, Chongqing, China; 2 Department of Pathology, Chongqing Medical University, Chongqing, China; 3 The First Affiliated Hospital, Chongqing Medical University, Chongqing, China; 4 Stem Cell Biology and Therapy Laboratory, The Children's Hospital, Chongqing Medical University, Chongqing, China; 5 Molecular Oncology Laboratory, Department of Surgery, The University of Chicago Medical Center, Chicago, Illinois, United States of America; Chinese University of Hong Kong, Hong Kong

## Abstract

**Background:**

The Hepatitis C virus (HCV) core protein has been implicated as a potential oncogene or a cofactor in HCV-related hepatocellular carcinoma (HCC), but the underlying mechanisms are unknown. Overactivation of the Wnt/β-catenin signaling is a major factor in oncogenesis of HCC. However, the pathogenesis of HCV core-associated Wnt/β-catenin activation remains to be further characterized. Therefore, we attempted to determine whether HCV core protein plays an important role in regulating Wnt/β-catenin signaling in HCC cells.

**Methodology:**

Wnt/β-catenin signaling activity was investigated in core-expressing hepatoma cells. Protein and gene expression were examined by Western blot, immunofluorescence staining, RT-qPCR, and reporter assay.

**Principal Findings:**

HCV core protein significantly enhances Tcf-dependent transcriptional activity induced by Wnt3A in HCC cell lines. Additionally, core protein increases and stabilizes β-catenin levels in hepatoma cell line Huh7 through inactivation of GSK-3β, which contributes to the up-regulation of downstream target genes, such as *c-Myc*, *cyclin D1, WISP2 and CTGF*. Also, core protein increases cell proliferation rate and promotes Wnt3A-induced tumor growth in the xenograft tumor model of human HCC.

**Conclusions/Significance:**

HCV core protein enhances Wnt/β-catenin signaling activity, hence playing an important role in HCV-associated carcinogenesis.

## Introduction

Hepatocellular carcinoma (HCC) ranks among the most common and deadly cancers worldwide [1]. HBV and HCV infection, alcohol abuse and exposure to aflatoxin B have been identified as major risk factors [2]–[4]. HCC associated with HCV infections evolves after many years of chronic infection and is generally preceded by the development of cirrhosis. However, the mechanisms underlying HCV-associated hepatocarcinogenesis are not fully understood.

HCV core is a 191 amino acid protein with RNA binding activity and may transactivate some transcription factors, such as NF-κB, AP-1 and SREBP [5]–[7]. It has been reported that HCV core protein may directly modulate hepatocyte proliferation and transformation through regulating important several signaling pathways [8]–[10], suggesting that HCV core protein may play an important role in the pathogenesis of HCV-induced neoplastic transformation of hepatocytes. However, molecular mechanism underlying HCV-induced neoplastic transformation remains to be thoroughly elucidated.

Aberrant activation of Wnt/β-catenin signaling results in enhanced cell growth and malignant cellular transformation. Inactivating mutations of *APC*, oncogenic mutations of β-catenin, upregulation of Frizzled type receptors (Fzds), and/or other Wnt signaling pathway alterations, play important roles in more than 33–67% of HCCs [11]–[14]. Fukutomi [15] recently reported that HCV core protein can up-regulate Wnt-1 and WISP-2 expression in Huh7 cells, leading to an increased cell proliferation, DNA synthesis and cell cycle progression. These findings suggest that activation of the Wnt/β-catenin signaling pathway may contribute significantly to the hepatocellular carcinogenesis.

In this study, we sought to investigate whether HCV core protein exerts any effect on Wnt/β-catenin signaling pathway and hence is involved in HCV-induced liver pathogenesis. We found that HCV core protein acts synergistically with Wnt3A on β-catenin-dependent transcriptional activity in HCC cell lines, and co-expression of core and Wnt3A induces stabilization and nuclear translocation of β-catenin, contributing to up-regulation of Wnt target gene expression. Cells transduced with both core and Wnt3A exhibit rapid proliferation, enhanced cell cycle progression. Co-expression of core and Wnt3A in HCC cells leads to an accelerated tumor formation in athymic nude mice. Thus, these data strongly suggested that Wnt/β-catenin signaling may be potentiated by core protein and hence play an important role in HCV pathogenesis.

## Materials and Methods

### Cell culture and chemicals

HEK293 cells [16] and human HCC lines HepG2, Huh7 [17] and SMMC-7721 [18] were maintained in complete Dulbecco's Modified Eagle's medium (DMEM) supplemented with 10% fetal bovine serum (FBS, Hyclone), 100 units/ml penicillin, and 100 µg/ml streptomycin at 37°C in 5%CO_2_. Human colon cancer cell line HCT116 [16] and Mouse hepatic progenitor cell line HP14.5 that was derived from mouse E14.5 fetal liver were also used in this study [19]–[20]. Unless indicated otherwise, all chemicals were purchased from Sigma-Aldrich.

### Construction of recombinant adenoviruses expressing HCV core and Wnt3A

For generating adenoviral vector expressing HCV core protein, the full-length of HCV core protein (genotype 1a) was PCR amplified from plasmid H/FL (kindly provided by Dr. Charles M. Rice of Rockefeller University, USA) [21] and subcloned into the shuttle vector pAdTrack-TO4. The adenoviral recombinant pAd-Core was subsequently generated and amplified in HEK293 cells using the AdEasy system [22]. Adenovirus expressing Wnt3A, namely AdWnt3A, was generated previously using the AdEasy system [16], [19]. In addition to the expression of transgenes, both Ad-Core and AdWnt3A also express GFP as a marker for monitoring infection efficiency. An analogous adenovirus expressing only GFP (AdGFP) was used as a control [16], [23].

### Preparation of Wnt3A conditioned medium

Wnt3A conditioned medium was prepared as described [16]. Briefly, subconfluent HCT116 cells (in 75 cm^2^ flaks) were infected with an optimal titer of AdWnt3A or AdGFP control. At 15 hr post-infection, the culture medium was changed to serum-free DMEM. Conditioned medium was collected at 48 hr after infection and used immediately.

### Luciferase assay

Cells were seeded in 25 cm^2^ cell culture flasks and transfected with 2 µg per flask of β-catenin/Tcf4-responsive luciferase reporter, pTOP-Luc [16], [22]–[23] using Lipofectamine 2000 (Invitrogen) according to the manufacturer's instructions. A *Renilla* luciferase reporter plasmid (Promega) was mixed as an internal control. At 16 hr after transfection, cells were replated to 24-well plates. At 4 hr after replating, cells were infected with AdGFP, Ad-Core, AdWnt3A, AdWnt3A plus Ad-Core or AdWnt3A plus AdGFP. At 24 hr after infection, cells were lysed and subjected to luciferase assays using Promega's Dual Luciferase Kit. Each assay condition was performed in triplicate.

### Immunofluorescence staining

Cells were fixed with 4% formaldehyde, permeabilized with 0.5% Triton, and followed by incubating with an anti-β-catenin antibody (Santa Cruz, sc-7199) overnight. After being washed, cells were incubated with Alexa Fluor 594-labeled secondary antibody (Invitrogen) for 45 min. Cells were counterstained with DAPI to label nuclei. The presence of β-catenin was visualized under a confocal immunofluorescence microscope.

### Western blotting analysis

Nuclear and cytoplasmic proteins were separated using Pierce's NE-PER Nuclear and Cytoplasmic Extraction Reagent according to the manufacturer's instructions. Whole cell extracts of exponentially growing cells were collected in lysis buffer (Promega) containing the complete cocktail of proteases inhibitors (Roche). Protein concentrations were determined by using the BCA protein assay reagent (Pierce). Approximately 50–70 µg of proteins were resolved on 10% polyacrylamide gels and electrotransferred to PVDF membranes (Millipore). The blots were probed with antibodies against β-catenin (Santa Cruz, sc-7199), HCV core (Abcam, ab2740), Wnt3A ( Cell Signaling, #2391), c-Myc (Santa Cruz, sc-764), cyclin D1 (Santa Cruz, sc-753), GSK3β (Santa Cruz, sc-9166) and phosphor (Ser9)-GSK3β (Cell Signaling, #9323). Secondary antibodies coupled to HRP were purchased from Jackson ImmunoResearch Laboratories. Proteins of interest were detected with Super Signal West Pico Chemiluminescent substrate Kits (Pierce).

### RNA isolation and quantitative RT-PCR analysis

Total RNAs were extracted from cultured cells using TRIZOL Reagents (Invitrogen) according to the manufacturer's protocol. Total RNA was used to generate cDNA templates by RT reaction with random hexamer and MMLV RT (Promega). The cDNA products were further diluted 5- to 10-fold and used as qPCR templates. The primer sequences used to detect the expression of HCV core, *c-Myc*, *cyclin D1*, *CTGF* and *WISP2* are listed in Table 1. Endogenous GAPDH expression was used as a normalization control. SYBR Green-based qPCR analysis was carried out using the DNA Engine Opticon 2 real-time PCR detection system (Bio-Rad, CA, USA). Duplicate reactions were carried out for each sample, and all samples were normalized by the expression level of GAPDH.

**Table 1:** Primer Sequences for RT-PCR.

Primers		Nucleotide sequence	Expected size(bp)
**HCV core**	Forward	5′-AGGGGCCCTAGATTGGGTGT-3′	186
	Reverse	5′-ACGGGGAGACAGGAGCCATC-3′	
**human Cyclin D1**	Forward	5′-GCCAGAGGCGGAGGAGAACA-3′	191
	Reverse	5′-AAGCGTGTGAGGCGGTAGTA-3′	
**human c-Myc**	Forward	5′-AGAGAAGCTGGCCTCCTACC-3′	244
	Reverse	5′-CGTCGAGGAGAGCAGAGAAT-3′	
**human CTGF**	Forward	5′-CCGTACTCCCAAAATCTCCA-3′	211
	Reverse	5′-GTAATGGCAGGCACAGGTCT-3′	
**human WISP2**	Forward	5′-CTGTATCGGGAAGGGGAGAC-3′	246
	Reverse	5′-GGAAGAGACAAGGCCAGAAA-3′	

### MTS proliferation assay

MTS assays were performed with the Cell Titer 96 AQ One Solution Cell Proliferation Assay (Promega). 20 ul MTS reagent was added to each well of the 96 well assay plate and incubated the plate for 2 hr at 37°C in 5% CO_2_. The absorbance at 490 nm was recorded every 24 hr in triplicate until day 4 by using a microplate reader (Synergy HT Multi-mode Microplate Reader, Bio-Tek). Each condition was repeated at least three times.

### Crystal violet cell viability assay

The crystal violet staining procedure was carried out as described [24]–[25]. Briefly, cells were fixed in 10% buffered formalin for 20 min and then stained with 0.5% crystal violet solution at room temperature for 30 min. The plates were washed and allowed to air dry and incubated with 0.2% Triton X-100 for 30 min at room temperature to dissolve the dye. Then, 100 µl samples from each well were transferred into 96-well microplates and read the absorbance at 570 nm in the microplate reader Synergy HT Multi-mode Microplate Reader (Bio-Tek).

### Cell cycle analysis

Cells were prepared for cell cycle analysis as described [26]. Briefly, cells were collected and fixed with 70% ethanol at 4°C. For flow cytometric assay, cells were washed with PBS and resuspended in propidium iodide (PI) staining solution. Samples were processed by using the FACSVantage SE (Becton Dickinson). The acquired data were analyzed using the CellQuest software (Becton Dickinson).

### Xenograft tumor model of human HCC in athymic nude mice

The care and use of experimental animals was in compliance with the institutional guidelines approved for our study. Athymic nude mice (4–6 week old, male, 18–25 g) were used for the studies. Huh7 cells were infected with AdGFP control, Ad-Core, AdGFP plus AdWnt3A, or Ad-Core plus AdWnt3A for 15 hr, and collected for subcutaneous injection (5×10^6^/injection) into the flanks of athymic nude (nu/nu) mice (*n* = 6 mice per group). At 6 wk after implantation, animals were sacrificed, and tumor masses were retrieved for histological analysis.

### Immunohistochemical staining

Retrieved xenograft samples were fixed with 4% paraformaldehyde, embedded and sectioned. Sections were incubated with β-catenin, c-Myc, cyclin D1 (Santa Cruz) or Ki67 (Santa Cruz, sc-23900) antibodies. Subsequently, the slides were incubated with EnVision System-HRP (DakoCytomation) and visualized using DAB substrate (DakoCytomation).

### Statistical analysis

The data were presented as mean ± SD. Statistical analysis was performed using the SPSS 15.0 software. Differences between groups were compared by the ANOVA test. In all assays, the probability value (*p*) of <0.05 was considered statistically significant. Error bars represent SDs of three independent experiments.

## Results

### HCV core protein plays an important role in activating Tcf-dependent transcriptional activity in hepatoma cells

In order to study the biological functions of HCV core protein in HCC cells, we first sought to construct and generate a recombinant adenovirus Ad-Core to effectively express core protein in target cells. As shown in Fig. S1, Ad-Core can effectively infect hepatocytes and express the transgene.

**Figure 1:**
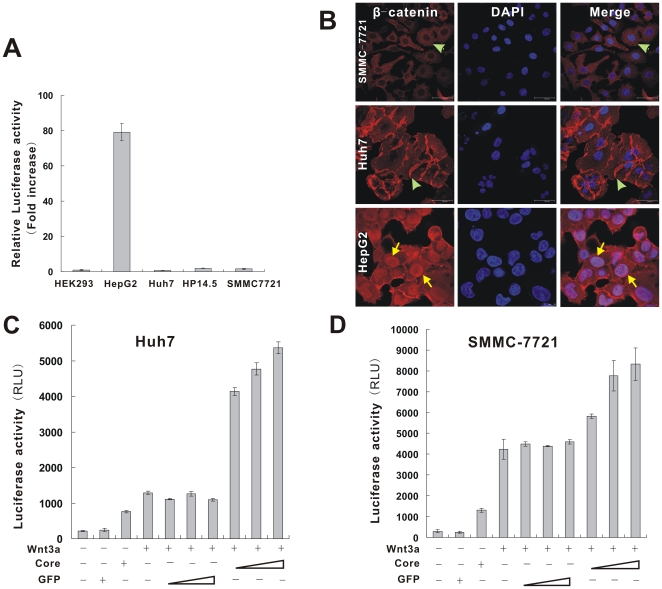
HCV core potentiates Wnt3A-induced TOP-Luc activity in hepatoma cells. (**A**) Endogenous Tcf-4-dependent transcriptional activity in different hepatoma cell lines. HEK293, mouse hepatic progenitor cells (HP14.5) and hepatoma cell lines (HepG2, Huh7, SMMC-7721) were transfected with Tcf4/LEF1 reporter pTOP-Luc. At 24 hr after transfection, cells were collected for luciferase assays. Data were present as mean ± S.D. Fold activation was calculated by dividing the relative luciferase activity of hepatic lines' with that of HEK293 cells'. (**B**) Cellular localization of β-catenin in SMMC-7721, Huh7 and HepG2 cells. Subconfluent cells were fixed and stained with an anti-β-catenin antibody (Santa Cruz Biotechnology) and a fluorescence-labeled secondary antibody. Cells were counterstained with 4′, 6-diamidino-2-phenylindole (DAPI) to label nuclei. The presence of β-catenin was visualized under a confocal immunofluorescence microscope. Membrane or cytoplasm stains were indicated by green arrows and nuclear stains were indicated by yellow arrows. (**C**) (**D**) HCV core activated Tcf-4-dependent transcription in Huh7 and SMMC-7721 cells. Cells were transfected with pTOP-Luc and then infected with AdGFP control, Ad-Core, AdWnt3A, AdWnt3A plus AdGFP or AdWnt3A plus Ad-Core. At 24 hr post infection, cells were collected for luciferase assays. Data are present as means ± S.D.

To determine the effects of HCV core protein on Wnt/β-catenin signaling, we then analyzed the endogenous β-catenin/Tcf activity in hepatoma cells by using pTOP-Luc lucifearse reporter assay. As shown in Fig. 1A, endogenous reporter activity was maintained at a basal level in Huh7, SMMC-7721 and mouse hepatic progenitor cell line HP14.5 [19]–[20], whereas Wnt signaling is already activated in HepG2 cells which harbor an oncogenic β-catenin mutation [27].

Immunostaining analysis indicated β-catenin was localized predominantly to cytoplasm and cytoplasmic membrane in SMMC-7721 and Huh7 cells. In contrast, in HepG2 cells nuclear staining of β-catenin was apparently observed with high cytoplasmic β-catenin accumulation (Fig. 1B). These results indicate that the basal β-catenin/Tcf activity is low in Huh7 or SMMC-7721 cells.

We next examined whether HCV core protein can potentiate Wnt/β-catenin signaling activity by measuring Tcf-4 dependent transcriptional activity. As shown in Fig. 1C and D, exogenous expression of Wnt3A or core alone exhibited limited activation of the TOP-Luc reporter in Huh7 and SMMC-7721 cells. And these findings are supported by an early report [27]. However, when Ad-Core and AdWnt3A were co-infected into Huh7 or SMMC-7721 cells, Wnt3A-induced Tcf-4 dependent transcriptional activity was enhanced by core protein in a dose-dependent manner, whereas the GFP control had no synergistic effect on Wnt3A-induced transcriptional activity. These results suggest that ectopic expression of HCV core can potentiate Wnt3A-induced TOP-Luc activity in Huh7 and SMMC-7721 cells.

### HCV core protein increases and stabilizes β-catenin levels in hepatoma cells through inactivation of GSK-3β

**Figure 2:**
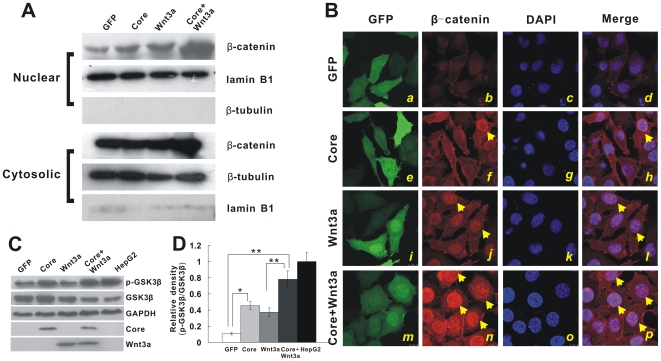
HCV core increases β-catenin levels in nuclear fractions through inactivation of GSK3β in Huh7 cells. (**A**) β-catenin levels in the nuclear fractions of Ad-Core-infected Huh7 cells. Subconfluent Huh7 cells were infected with Ad-Core, AdGFP, AdWnt3A or Ad-Core plus AdWnt3A for 24 hr. Both cytosolic and nuclear fractions prepared from cells were immunoblotted with anti-β-catenin antibody. The subcellular fractions were verified by immunoblotting using either anti-β-tubulin or lamin B1 antibody. (**B**) Nuclear localization of β-catenin induced by HCV core and Wnt3A co-infection. SMMC-7721 cells were infected with AdGFP (panels ***a*** to ***d***), Ad-Core (panels ***e*** to ***h***), AdWnt3A (panels ***i*** to ***l***) or Ad-Core plus AdWnt3A (panels ***m*** to ***p***) for 24 hr. Cells were analyzed for immunofluorescence staining as described in **Figure 1**. Nuclear translocation of β-catenin was indicated by yellow arrows. (**C**) Huh7 cells were infected with AdGFP, Ad-Core, AdWnt3A or Ad-Core plus AdWnt3A for 36 hr. Total cell lysate was subjected to immunoblotting with anti-phosphor-GSK3β, anti-GSK3β, anti-HCV core or anti-Wnt3A antibody. GAPDH was used as a loading control (bottom panel). Data were obtained from three independent experiments, and representative results are shown. (**D**) Quantitative analysis of the band intensity in Fig. 2C using the ImageJ software. * *p*<0.05; **p<0.01.

The hallmark of canonical Wnt signaling activation is cytoplasmic and nuclear accumulation of stabilized β-catenin protein. To examine the subcellular distribution of β-catenin in hepatoma cells producing core protein, we infected Huh7 cells with Ad-Core, AdGFP, AdWnt3A or Ad-Core plus AdWnt3A, and then detected β-catenin expression level in the cytoplasmic and nuclear fractions. As expected, co-expression of HCV core and Wnt3A induced β-catenin accumulation both in nuclear and cytosolic fraction. However, apparent nuclear translocation of β-catenin was not examined in GFP control cells (Figure 2A).

To corroborate these findings, we next analyzed cellular localization of β-catenin in SMMC-7721 cells by immunostaining. Cells were infected with a comparable titer of AdGFP, Ad-Core, AdWnt3A or AdWnt3A plus Ad-Core (Figure 2B, panels ***a***, ***e***, ***i*** and ***m***. GFP fluorescence shows infected cells). Consistent with the immunoblotting results, partially nuclear translocation of β-catenin was observed when cells were infected with Ad-Core but not GFP control (Figure 2B, panels ***b*** to ***d***; ***f*** to ***h***). This nuclear translocation effect was further strengthened when core and Wnt3A were co-expressed (Figure 2B, panels ***n*** to ***p***), Similar results for nuclear translocation of β-catenin induced by HCV core were obtained in hepatoma cell lines Huh7 cells using immunostaining assay (Fig. S2).

In order to eliminate possible interference caused by adenovirus co-infections, we further examined the subcellular distribution of β-catenin in core-expression cells by using Wnt3A conditioned medium instead of adenovirus co-infections. Briefly, Huh 7 or HepG2 cells were infected with AdCore or AdGFP for 24 h, and then stimulated with Wnt3A conditioned medium for 0, 2 and 4 h. The cytoplasmic and nuclear fractions were prepared for immunoblotted with an anti-β-catenin antibody. We found that β-catenin levels were significantly increased in the nuclear fraction of core-expression hepatoma cells, peaked at 4 hrs after Wnt3A stimulation, corresponding to a simultaneous decrease of β-catenin protein level in the cytoplasmic fraction (Fig. S3). Taken together, these results suggest that HCV core may induce β-catenin accumulation and nuclear translocation, thus leading to the activation of Tcf4-dependent transcriptional regulation.

We next investigated the possible mechanism through which core protein induces β-catenin accumulation. Phosphorylation of β-catenin by GSK-3β causes its degradation through the ubiquitin-proteasome system. We examined whether accumulation and stabilization of β-catenin in core-expressing cells was resulted from inactivation of GSK3β. Since β-catenin is mutated and partially localized in the nucleus of HepG2 cells (Figure 1B and Fig. S3B), this cell line is a suitable negative control to elucidate the role of GSK3β inactivation in core-expression cells. Not surprisingly, the ratio of p-GSK-3β vs. total GSK-3β was much higher in HepG2 cells than that of Huh7 cells at basal condition (Figure 2C). Phosphorylation of GSK-3β at the Ser-9 residue in HCV core-expressing Huh7 cells was much stronger than that in GFP control cells (Figure 2C, Top panel). Quantitative analysis indicated that p-GSK-3β expression level in core-expressing cells was nearly 3-fold of GFP control's. The increased phosphorylation of GSK3β was further potentiated when cells were co-infected with Ad-Core and Ad-Wnt3A (Figure 2D, *p*<0.05). It is well established that Ser-9 phosphorylation inactivates GSK-3β kinase activity [28]. Thus, these data strongly suggest that HCV core may up-regulate β-catenin through inactivation of GSK-3β by phosphorylation at serine-9.

### Additive effect of HCV core protein on up-regulation of Wnt/β-catenin downstream target genes

**Figure 3:**
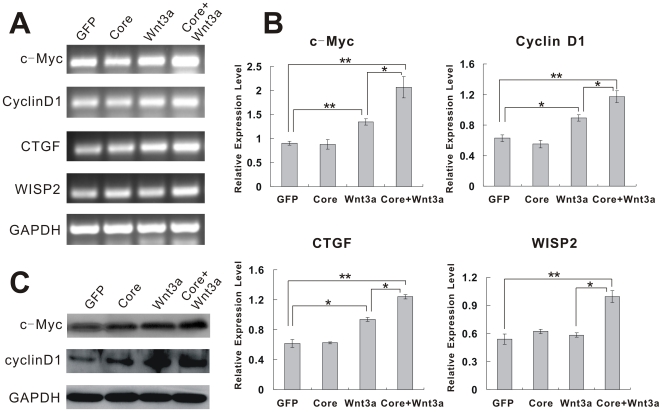
HCV core enhances Wnt3A-induced target genes expression. (**A**) Semi-quantitative RT-PCR analysis of the mRNA expression for known Wnt target genes after ectopic expression of HCV core and/or Wnt3A. Huh7 cells were infected with Ad-Core, AdWnt3A, AdGFP control or Ad-Core plus AdWnt3A for 36 hr. Total RNA was isolated for RT-PCR analysis using primers specific for indicated genes. Data were obtained from three independent experiments, and representative results are shown. All samples were normalized with GAPDH. (**B**) Quantitative analysis of target gene mRNA expression level. The expression level of c-Myc, cyclinD1, CTGF and WISP2 were detected by qRT-PCR. Data are shown as the means ± S.D from three independent experiments. * *p*<0.05; **p<0.01. (**C**) HCV core enhances Wnt3A-induced downstream genes at protein level. Cells were treated as described in **Figure 3A**, and then total cell lysate was prepared at 48 hr after infection. C-Myc and cyclin D1 expression was assessed by Western blotting using respective antibodies. GAPDH was used as a loading control. Data were obtained from three independent experiments, and representative results are shown.

The activation of Wnt/β-catenin signaling pathway leads to transcriptional regulation of Wnt-responsive genes, which regulate cell proliferation and cell migration [15], [29]. The *c-Myc*, *cyclin D1*, *CTGF* and *WISP2* are well-known target genes of canonical Wnt signaling [22], [30]–[31], and some of them are known to be over-expressed in HCC tissues [32]. Next we tested whether HCV core could up-regulate Wnt signaling in Huh7 cells. As shown in Fig. 3A and 3B, when cells were co-infected with Ad-Core and AdWnt3A, mRNA expression levels of c-Myc, cyclin D1, CTGF and WISP2 were approximately 1.7-, 1.5-, 1.2- and 1.8-fold higher than that in AdWnt3A-infected cells (*p*<0.05). Western blot analysis indicated that the protein expression of c-Myc and cyclinD1 were further elevated in these cells than in Wnt3A-treated cells (Fig. 3C). Thus, these findings, together with the results from Tcf4- reporter assays and nuclear β-catenin localization experiments, have demonstrated that exogenous expression of HCV core in Huh7 cells enhances Wnt3A-induced Tcf-dependent transcriptional activity, promotes β-catenin nuclear translocation, and additively up-regulates the expression of Wnt-target genes, such as *c-Myc* and *cyclin D1*.

### HCV core increases cell proliferation rate and promotes cell cycle progression

**Figure 4:**
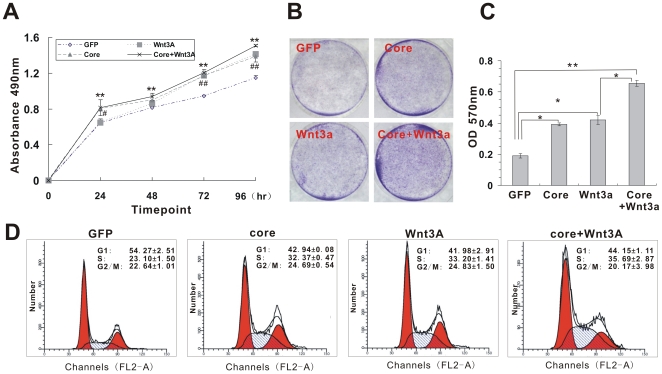
HCV core promotes cell proliferation in Huh7 cells. (**A**) Cell proliferation curves. Huh7 cells were infected with an optimal titer of Ad-Core, AdWnt3A, AdGFP control or Ad-Core plus AdWnt3A and then plated into 96-well plate at 0.5×10^4^/well. Cells were counted every 24 hr in triplicate. Data are present as means ± S.D. ** *P*<0.01 (Core plus Wnt3A vs GFP). **#** *P*<0.05; ## *P*<0.01 (Core vs GFP). (**B**) Crystal violet cell viability assay of core-expressing Huh7 cells. Huh7 cells were infected with Ad-Core, AdWnt3A, AdGFP control or Ad-Core plus AdWnt3A, and then plated at low density in 6-well plate. The cells were stained with crystal violet at 4 days after plating. Data were obtained from three independent experiments, and representative results are shown. (**C**) Cell viability was quantitatively assessed using a colorimetric assay (see Methods). Differences in colorimetric values were subjected to statistical analysis. *P<0.05; ** *P*<0.01. (**D**) Flow cytometric cell cycle analysis. Huh7 cells were infected with Ad-Core, AdWnt3A, AdGFP control or Ad-Core plus AdWnt3A. At 120 hr post-infection, cells were collected, fixed, incubated with the propidium iodide (PI)/RNase staining buffer, and subjected to flow cytometry. G1, S, and G2/M populations are indicated as percentages of the whole population. Data were expressed in means ± S.D from at least three independent experiments.

We next examined the effect of HCV core protein on biological functions of Wnt/β-catenin signaling. As shown in Figure 4A, over-expression of HCV core alone resulted in an increased proliferation rate in Huh7 cells. A similar growth-promoting activity by HCV core alone was observed by cell viability assay (Figure 4B). Moreover, cells co-infected with Ad-Core and AdWnt3A showed stronger proliferation capacity than AdWnt3A or Ad-Core-treated cells at 96 hr post infection (*p*<0.05). Quantitative viability analysis confirmed the additive effect of HCV core on Wnt3A-induced growth promotion (Figure 4C, *p*<0.05).

Furthermore, we determined whether cell cycle progression was affected by HCV core protein. The cell cycle analysis showed that the S-phase distribution in cells transduced with core, Wnt3A or core plus Wnt3A were 32.37%, 33.2% and 35.69% respectively (Figure 4D), much higher than that of GFP control (23.10%) (*p*<0.05). Quantitative cell cycle analysis from three batches of experiments showed ectopic expression of core, Wnt3A or core plus Wnt3A significantly promoted G1- to S-phase transition in Huh7 cells, especially at 72 hr and 120 hr post-infection (Table S1). Thus, HCV core can accelerate cell proliferation which may contribute to the increase in cell numbers in S-phase. These data were consistent with the results of core protein-promoted proliferation and viability assay. Taken together, the above data strongly suggest that HCV core may be involved in promoting the transition of cells from the G1-phase to S-phase of the cell cycle, consistent with the well-recognized growth-promoting activity of Wnt/β-catenin signaling.

### Core protein synergizes with Wnt/β-catenin signaling in promoting xenograft tumor growth

**Figure 5:**
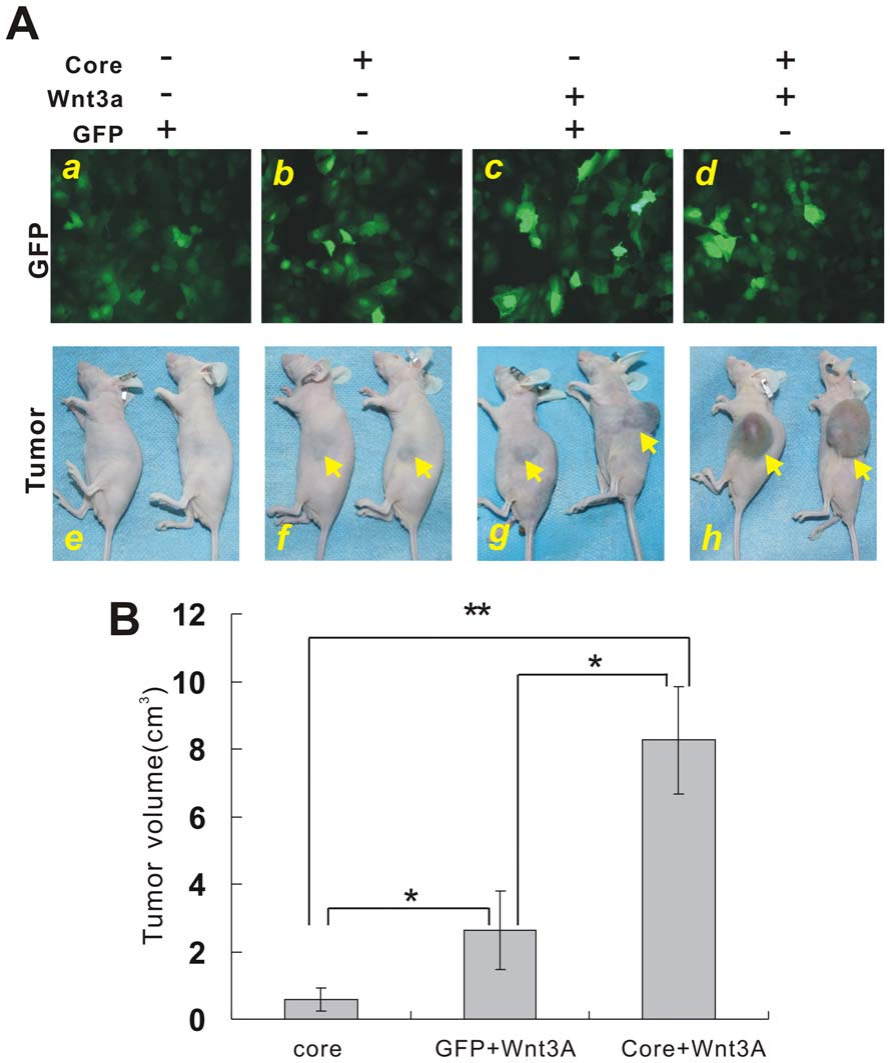
Over-expression of HCV core promotes Wnt3A-induced xenograft tumor growth in vivo. (**A**) Ectopic expression of HCV core in Huh7 cells accelerated Wnt3A-induced tumor growth. Huh7 cells were infected with AdGFP control, Ad-Core, AdGFP plus AdWnt3A or Ad-Core plus AdWnt3A for 15 hr, and the infection efficiency was examined under a fluorescence microscope (Panels ***a*** to ***d***). The infected cells were collected and subjected to subcutaneous injection into the flanks of athymic mice. At 6 weeks post implantation, animals were sacrificed and tumor masses were retrieved. Representative gross images of xenograft tumors are shown (Panels ***f*** to ***h***). Gross tumors are indicated by arrows. Note that GFP-treated Huh7 cells did not form any detectable tumor masses at the end point of the experiment (panel ***e***). (**B**) Comparison of tumor size in the xenograft model. The infected cells were treated as described in **Figure 5A**. Tumor sizes were measured at 6 wk after injection and subjected to statistical analysis. *P<0.05; ** *P*<0.01.

**Figure 6:**
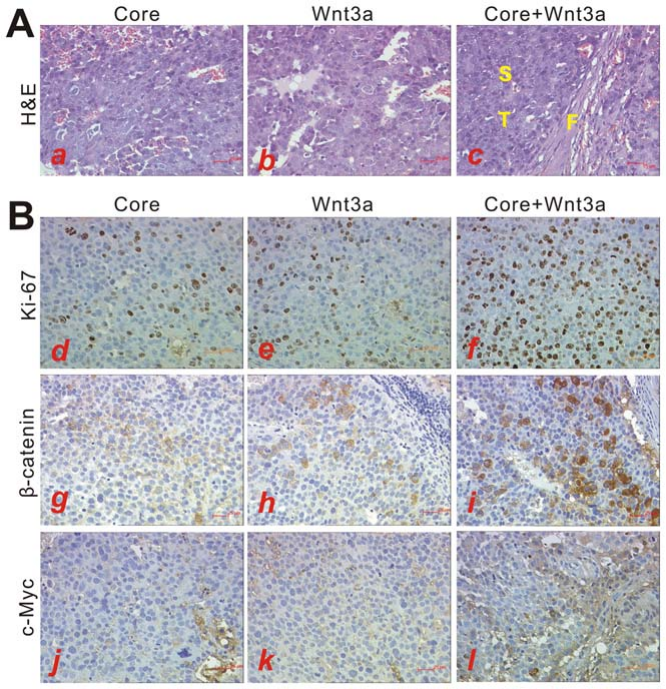
HCV core promotes cell proliferation in xenograft tumors. Retrieved tumor samples (from Figure 5) were fixed and subjected to histological evaluation. (**A**) Hematoxylin& eosin staining of tumors (panels ***a*** to ***c***), showing examples of tumor cells surrounded by fibrous connective tissue. S, Sinusoid. F, Fibrous connective tissue. T, tumor cells. Magnification,×400. (**B**) Immunohistochemical detection of Ki67 (***d*** to ***f***), β-catenin (***g*** to ***i***) and c-Myc (***j*** to ***l***) in xenograft tumor samples. The positive cells stained brown. Representative images are shown (Paraffin section; ×400).

We further investigated the tumorigenic activity in HCC cells co-infected with core and Wnt3A. Huh7 cells were infected with a comparable titer of AdGFP, Ad-Core, AdWnt3A plus AdGFP or AdWnt3A plus Ad-Core (Figure 5A), and then injected into nude mice subcutaneously. All mice injected with core, Wnt3A plus GFP or Wnt3A plus core formed xenograft tumors within 6 weeks after inoculation, and tumor sizes in mice injected with Wnt3A and core co-infected cells were much larger than that of either core or Wnt3A alone (Figure 5B, *p*<0.01). On the other hand, none of mice injected with GFP control showed tumor formation for up to 10 weeks, indicating that Huh7 cells are not highly tumorigenic under our experimental conditions. Hematoxylin & eosin staining of the retrieved tumor samples revealed that tumor cells with multinuclear and frequent mitosis were surrounded by fibrous connective tissues (Figure 6A, panels ***a*** to ***c***). Immunohistochemical (IHC) analysis with a Ki-67 antibody revealed that cell proliferation activities were increased in cells co-infected with Wnt3A plus HCV core. Meanwhile, strong β-catenin protein expression was observed in cytoplasm and whole cells (Figure 6B, panels ***d*** to ***f***; ***g*** to ***i***). We also examined the protein expression of Wnt/β-catenin targets c-Myc and cyclin D1 by IHC staining. As shown in Figure 6B, there was a dramatic increase in the expression level of c-Myc (panels ***j*** to ***l***) and cyclin D1 (data not shown) after co-expression of Wnt3A and core in hepatoma cells. Taken together, these in vivo results suggest that HCV core protein may potentiate Wnt/β-catenin-induced tumor growth in HCC xenograft model.

## Discussion

Although both virus-induced and immunologically-mediated hepatocytes damage play an important role in pathogenesis, the mechanisms underlying HCV persistence and pathogenesis have been poorly understood. HCV core protein has been reported to play a critical role in HCV-associated liver diseases by activation of several cellular signal transduction, direct interaction with proto-oncogenes [33]–[34], and to transform cell lines and induce tumors in transgenic mouse models [10], [35]–[37]. Recently, HCC-derived core protein has been shown to shift TGF-β responses from tumor suppression to epithelial-mesenchymal transition (EMT), which contributes to the promotion of cell invasion and metastasis [17].

In the present study, we sought to investigate whether HCV core protein directly activates Wnt/β-catenin signaling pathway and hence is involved in HCV-induced liver pathogenesis. We found that HCV core protein synergizes Wnt3A-mediated β-catenin-dependent transcriptional activity in hepatocytes and HCC cells. Moreover, co-expression of core and Wnt3A gene induces stabilization and nuclear translocation of β-catenin, contributing to the up-regulation of Wnt-target gene expression, such as *c-Myc* and *cyclin D1*. We further demonstrated that cells transduced with core and Wnt3A exhibited increased proliferation, promoted cell cycle progression and accelerated tumor formation in athymic nude mice. Our results are supported by several studies on interaction between virus-encoded proteins and Wnt/β-catenin tumorigenic signaling. For example, HCV NS5A protein was shown to activate β-catenin signaling cascades through increasing the stability of β-catenin [38]. HBV X protein along with Wnt-1 activated Wnt/β-catenin signaling in Huh7 cells [27].

The stabilization and accumulation of β-catenin can be achieved by two possible mechanisms. One mechanism is Wnt-dependent stabilization of β-catenin, which was modulated by several components upstream of β-catenin molecule [18]. The other mechanism is phosphorylation of GSK3β at the Ser9 residue. GSK3β is a critical component that controls the distribution balance of β-catenin between cytoplasm and nucleus. Inactivation of GSK3β leads to the accumulation of β-catenin in the cytoplasm. Many growth factors, including IGF-1, EGF and HGF have been implicated in GSK3β inhibition [39]–[41]. It has been reported that GSK3β activity can be modulated by virus-encoded proteins. For example, the latent membrane protein 2A of Epstein-Barr virus and Hepatitis B virus X protein have been shown to enhance β-catenin accumulation through Ser9 phosphorylation of GSK3β [27], [42]. Erk-mediated inactivation of GSK3β may be involved in HBx- and growth factor-induced β-catenin stabilization [28]. Recently, the nonstructural 5A (NS5A) protein of HCV has been shown to inactivate GSK3β activity and subsequently increase accumulation of β-catenin in hepatoma cells [38]. Here, we demonstrated that the phosphorylation of GSK3β was increased in core-expression cells, indicating core may up-regulate β-catenin through inactivation of GSK-3β by phosphorylation at Serine-9. Further studies should be directed to explore the molecular mechanism behind stabilization of β-catenin involving GSK3β phosphorylation.

Exogenous core expression was shown to enhance Wnt3A-stimulated HCC tumor growth, possibly by inducing β-catenin accumulation and oncogene overexpression. Interestingly, in xenogarft animal model nuclear translocation of β-catenin in tumor cells was less prominent. There are several possible explanations. First, previous data showed that neoplastic hepatoma cells with nuclear accumulation of β-catenin were restricted to the periphery of tumor nodules or were in small tumor regions detached from large nodules [43]. The liver tissue sampling we used may not include this region; therefore, nuclear accumulation of β-catenin can not be readily observed. Secondly, Huh7 cells were classified as “well-differentiated” hepatoma cells according to the epithelial gene expression profile [44], while nuclear accumulation of β-catenin was correlated with the dedifferentiation of tumor cells to immature hepatocyte progenitors [45]. Thus, the differentiation status of xenograft tumors may determine the subcellular distribution of β-catenin. Lastly some components upstream of β-catenin may be negatively modulated in Huh7 cells, leading to inhibition of β-catenin translocation. It is conceivable that some if not all of the above possibilities may be responsible for the β-catenin immunostaining results in xenograft tumors.

In summary, by co-expressing HCV core protein and Wnt3A in hepatocytes and HCC cell lines, we have found that the HCV core protein can potentiate and synergize with Wnt/β-catenin signaling pathway in promoting cell proliferation and tumor growth. These results strongly suggest that HCV core, possibly through synergizing Wnt-induced stabilization and accumulation of β-catenin, may play an important role in HCV pathogenesis. Nonetheless, the exact role of HCV core in Wnt/β-catenin-induced HCC tumorigenesis remains to be fully investigated. Ultimately, this line of investigation may lead to the development of novel anti-HCV therapies by targeting both HCV itself and the canonical Wnt signaling pathway.

## Supporting Information

Figure S1 **The recombinant adenovirus effectively expresses core protein in target cells.** (**A**) Effective infection of hepatocytes with Ad-Core. Huh7 or HP14.5 cells were infected with Ad-Core and the infection efficiency was examined under a fluorescence microscope. BF: blank field. Magnification, (400. (**B**) Ectopic expression of HCV core in Huh7 cells. Huh7 cells were infected with Ad-Core, AdWnt3A or AdGFP control for 36(hr. Total RNA was isolated for RT-PCR analysis using primers specific for HCV core gene (a), and protein expression was determined by Western blotting using anti-core antibody (Abcam) (b). Endogenous GAPDH expression was used as a control.
(TIF)

Figure S2 **HCV core increases the nuclear translocation of β-catenin induced by Wnt3A.** Huh7 cells were infected with AdGFP (panels *a* to *d*), Ad-Core (panels *e* to *h*), AdWnt3A (panels *i* to *l*) or Ad-Core plus AdWnt3A (panels *m* to *p*) for 24 hr. Cells were fixed and subjected to immunofluorescence staining as described in Figure 1. Nuclear translocation of β-catenin was indicated by yellow arrows.
(TIF)

Figure S3 **Ectopic expression of HCV core enhances nuclear accumulation of β-catenin in Huh7 and HepG2 cells.** Subconfluent hepatoma Huh7 (A) and HepG2 (B) cells were infected with Ad-Core or AdGFP for 24 hr, and stimulated with Wnt3A conditioned medium for 0, 2 and 4 hr. Both cytosolic (C) and nuclear (N) fractions were prepared and subjected to Western blotting analysis with an anti-β-catenin antibody. The subcellular fractions were verified by immunoblotting analysis using either anti-β-tubulin or lamin B1 antibody. WT, wild type; Mut, mutant β-catenin.
(TIF)

Table S1 **Cell Cycle (S Phase) analysis.**
(DOC)
