# Supplementary figures and images for: Enhancement of Canonical Wnt/β-Catenin Signaling Activity by HCV Core Protein Promotes Cell Growth of Hepatocellular Carcinoma Cells
Source: PLoS One. 2011 Nov 15;6(11):e27496. doi: 10.1371/journal.pone.0027496 (PMC3216985; doi:10.1371/journal.pone.0027496)

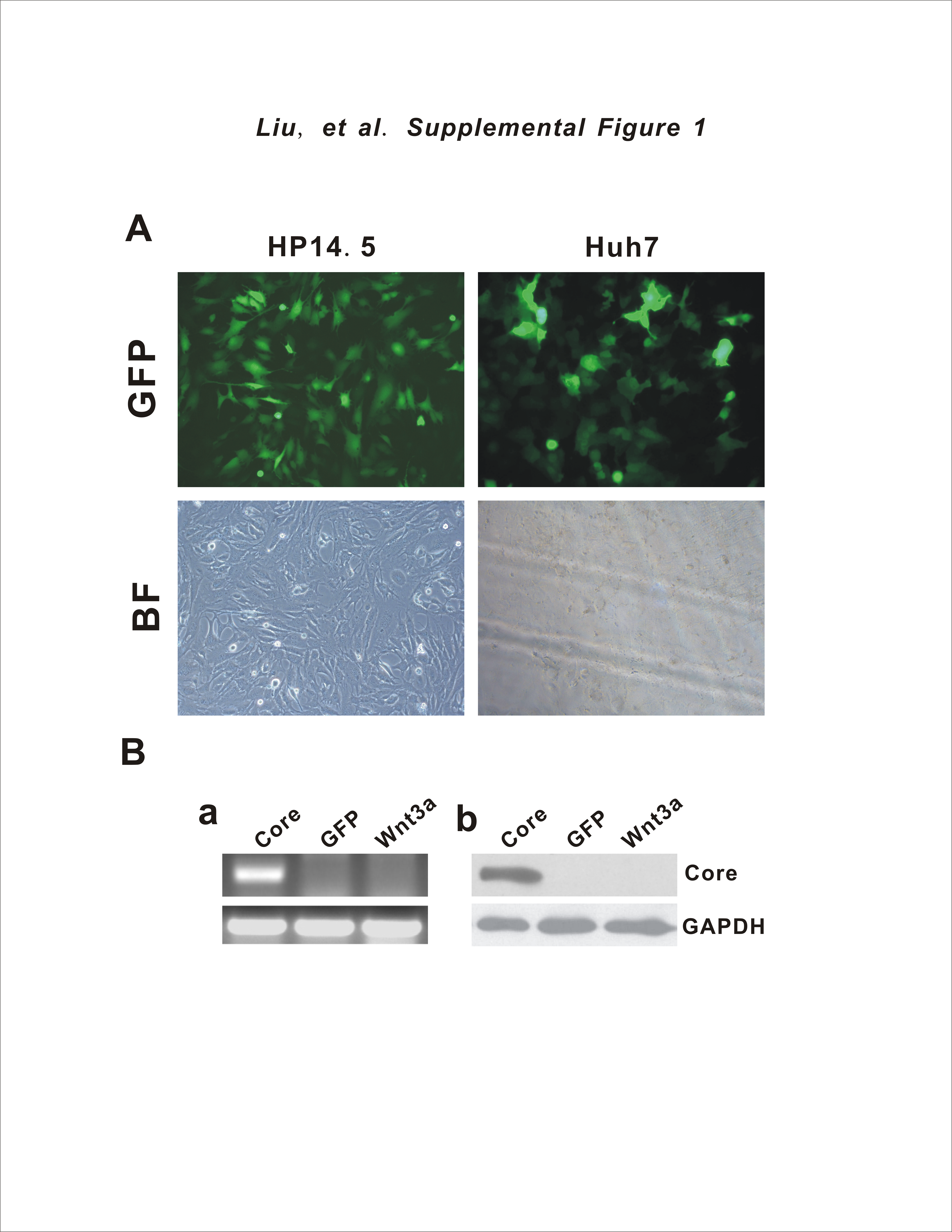

Supplement: Figure S1 — The recombinant adenovirus effectively expresses core protein in target cells. (A) Effective infection of hepatocytes with Ad-Core. Huh7 or HP14.5 cells were infected with Ad-Core and the infection efficiency was examined under a fluorescence microscope. BF: blank field. Magnification, (400. (B) Ectopic expression of HCV core in Huh7 cells. Huh7 cells were infected with Ad-Core, AdWnt3A or AdGFP control for 36(hr. Total RNA was isolated for RT-PCR analysis using primers specific for HCV core gene (a), and protein expression was determined by Western blotting using anti-core antibody (Abcam) (b). Endogenous GAPDH expression was used as a control. (TIF) [file pone.0027496.s001.tif]

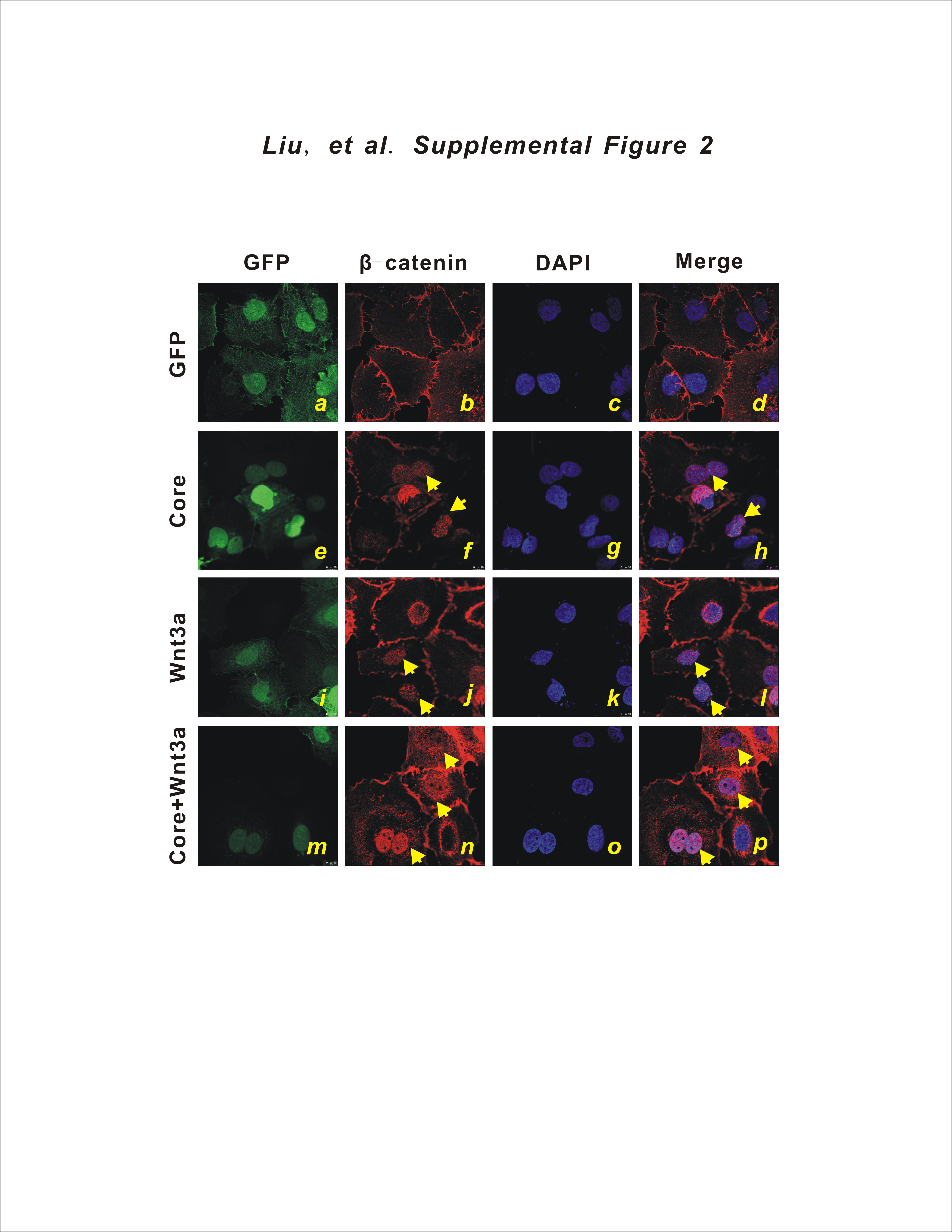

Supplement: Figure S2 — HCV core increases the nuclear translocation of β-catenin induced by Wnt3A. Huh7 cells were infected with AdGFP (panels a to d), Ad-Core (panels e to h), AdWnt3A (panels i to l) or Ad-Core plus AdWnt3A (panels m to p) for 24 hr. Cells were fixed and subjected to immunofluorescence staining as described in Figure 1. Nuclear translocation of β-catenin was indicated by yellow arrows. (TIF) [file pone.0027496.s002.tif]

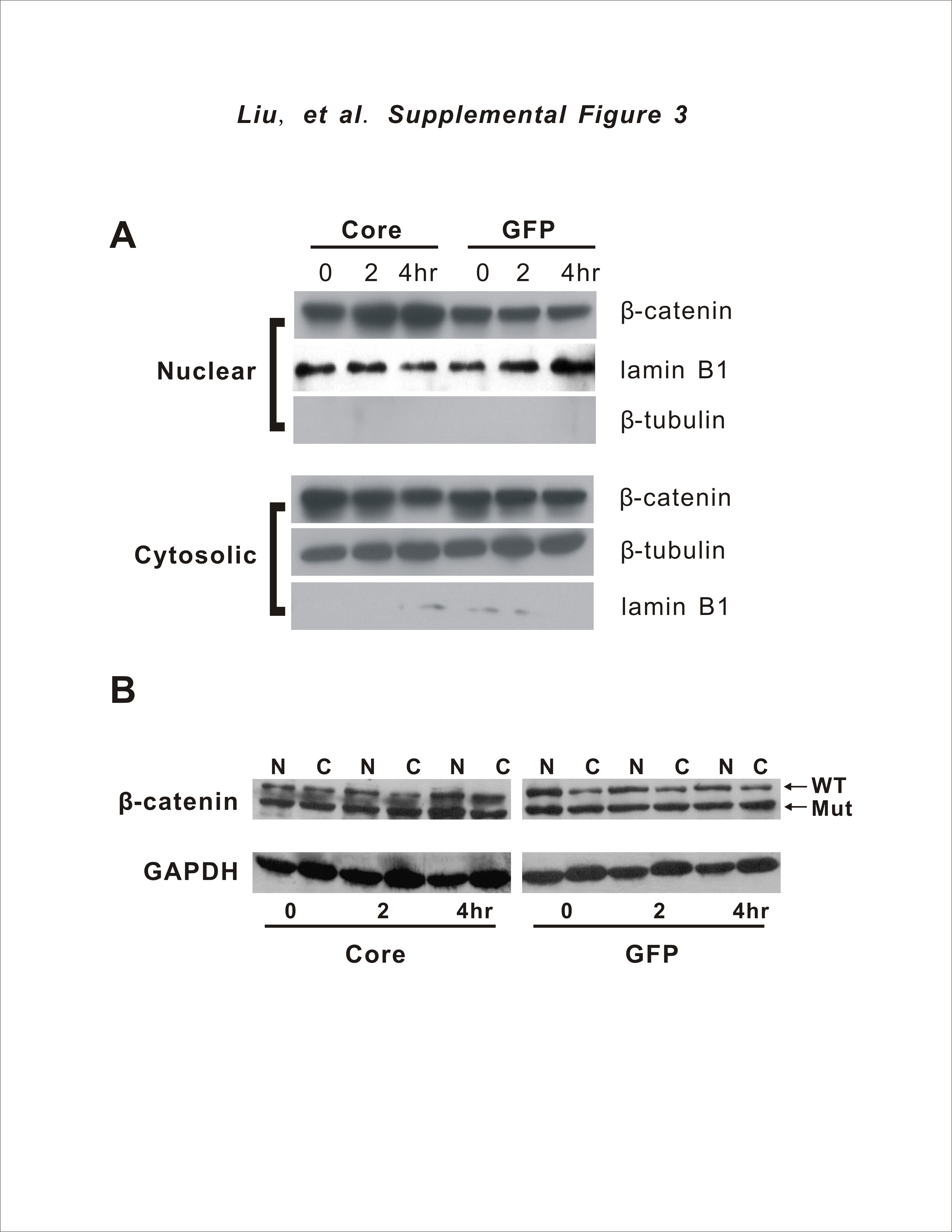

Supplement: Figure S3 — Ectopic expression of HCV core enhances nuclear accumulation of β-catenin in Huh7 and HepG2 cells. Subconfluent hepatoma Huh7 (A) and HepG2 (B) cells were infected with Ad-Core or AdGFP for 24 hr, and stimulated with Wnt3A conditioned medium for 0, 2 and 4 hr. Both cytosolic (C) and nuclear (N) fractions were prepared and subjected to Western blotting analysis with an anti-β-catenin antibody. The subcellular fractions were verified by immunoblotting analysis using either anti-β-tubulin or lamin B1 antibody. WT, wild type; Mut, mutant β-catenin. (TIF) [file pone.0027496.s003.tif]
