# Supplementary material for: Enhancement of Canonical Wnt/β-Catenin Signaling Activity by HCV Core Protein Promotes Cell Growth of Hepatocellular Carcinoma Cells
Source: PLoS One. 2011 Nov 15;6(11):e27496. doi: 10.1371/journal.pone.0027496 (PMC3216985; doi:10.1371/journal.pone.0027496)
Supplement: Table S1 — Cell Cycle (S Phase) analysis. (DOC) [file pone.0027496.s004.doc]

**Table. S1. Cell Cycle (S Phase) analysis.**

|  | **24h** | **72h** | **120h** |
| --- | --- | --- | --- |
| **GFP** | 19.85±0.49 | 21.77±1.02 | 23.10±1.50 |
| **Core** | 20.27±1.87 | 31.66±2.01* | 32.37±0.47 ** |
| **Wnt3a** | 21.36±0.66 | 30.49±1.53* | 33.20±1.41 ** |
| **Core+Wnt3a** | 27.49±1.88* | 33.79±2.47* | 35.69±2.87 ** |
| ***Note: * p<0.05,** p<0.01*** | | | |

Huh7 cells were infected with AdGFP control, Ad-Core, AdWnt3A or Ad-Core plus AdWnt3A. At 24 hr, 72 hr, and 120 hr post-infection, cells were collected and subjected to flow cytometry. Percentage of cells in S phase was statistically analyzed, **P*<0.05; ** *P*<0.01. (vs GFP control). Each assay condition was done in triplicate.
